# Supplementary material for: Probiotics-Fermented Grifola frondosa Total Active Components: Better Antioxidation and Microflora Regulation for Alleviating Alcoholic Liver Damage in Mice
Source: Int J Mol Sci. 2023 Jan 11;24(2):1406. doi: 10.3390/ijms24021406 (PMC9862899; doi:10.3390/ijms24021406)
Supplement: Supplementary file 1 [file ijms-24-01406-s001.zip › ijms-2082052-supplementary.pdf]

Table S1. The relative abundance of intestinal microbiota at phylum. Data are expressed as the mean ± SD (n = 5)

Taxonomy	NC	EtOH	P	GF	FGF
Firmicutes	22239.2±4564.9	23980.8±2032.3	20065.4±4240.9	17401±2892.6	18727.4±2715.8
Bacteroidetes	19246±2116.5	16170.6±1144.6	18023±2950.2	18966.2±1972.1	19761.2±928.9
Proteobacteria	4294.2±3186	2402.4±115.9	1941.2±845.1	1071±492.1	2832.8±1884.3
Actinobacteria	1332.2±302.5	1965.4±474.1	1866.2±949.2	1401.6±680.9	1083.4±214.9
Tenericutes	142.8±101.9	295.4±323.2	266.2±261.8	77.6±38.7	61.8±26.1
Acidobacteria	203.8±72.5	273.4±104.9	198.6±139.7	0.6±0.8	49.4±68.1
Others	3512.8±1009.2	1827.6±654.2	3577.2±1654.2	4181.4±1577	5261.4±3222.8

Table S2. The relative abundance of intestinal microbiota at genus. Data are expressed as the mean ± SD (n = 5)

Taxonomy	NC	EtOH	P	GF	FGF
Faecalibaculum	114.6±18.6	446.8±52.9	1377±360.6	3777±1284.1	314.8±127.2
Lactobacillus	1904.2±116.4	3150±217.8	4679.8±299.3	2223.2±1016.1	4692.8±370.4
Dubosiella	1775.8±450	1852.2±1116.7	3948.6±1401.5	1811.4±529.2	1708.6±608.3
Bacteroides	1246.4±203.6	1644.8±829.7	496.6±200.8	463.4±119.8	237±5.8
Alloprevotella	579.6±79.8	700±277	472±180.6	3274.2±2026.6	1264±163.8
Desulfovibrio	996.8±269.8	1319.2±285.3	347.2±114.8	656.2±34.6	913.8±225.4
Candidatus_Saccharimonas	461.6±72.7	184±85.8	527.2±302.7	340.6±115.2	602.6±317.1
Comamonas	81.8±24	65.8±20.2	0.6±1.2	0±0	0±0
unidentified_Ruminococcaceae	1628.2±371.4	764.8±244.9	1112.8±213.8	926.6±268.9	1645.4±289.6
Bifidobacterium	292±47.6	340.4±177.7	1071.2±372.6	1195.6±91.1	284.8±82.1
Citrobacter	53.4±5.5	54.6±18.3	1±1.3	0±0	0.2±0.4
Alistipes	1109.4±120	460.8±71.2	328.8±86.4	574.6±96.6	942.4±64.4
Enterorhabdus	314.6±50.5	287.6±120.9	248.6±101.5	333.4±74.8	587±91.8
Ruminiclostridium	813.8±154.8	740.2±119.4	547.8±153.7	405±149	649±157
Blautia	141.2±49.5	170.2±40.9	117±62.2	138.6±25.7	48.6±6.9
Intestinimonas	245.4±62.8	227.2±92.7	300±75.5	155.8±63.1	195±78.2
Parabacteroides	115.8±24.6	242±62.7	117.6±26.4	205.8±16.5	311.8±35.3
Oscillibacter	415.8±136.9	439.6±69.5	285.4±111.5	173.6±69.6	300.4±78.5
Roseburia	315±33.3	222±38.9	158.2±61.1	99.2±12.7	158.8±38.7
Odoribacter	568±107.6	23.2±9.2	26±13.2	2.8±2	38.8±29
Anaerotruncus	266.2±39.8	154.8±33.4	146.2±21.7	204.6±49.7	240±100.7
Angelakisella	126.8±38	65.2±18.3	63.4±10.4	79.2±47.4	118.8±22.8
Turicibacter	207±58.6	56.8±16.6	30±9	25.4±5.9	48.2±11.3
Parasutterella	259±31.3	78.6±12.1	111.8±16.7	143.8±27.2	108.2±24
Bacillus	222.8±92	190.8±74.2	79.8±50.1	0±0	10±13.8
Lachnoclostridium	38.4±16.2	73.8±15.7	61±14.6	63.2±34.1	119.4±25.2
Akkermansia	2.6±3.8	1.2±1.5	10.6±10.1	3.4±4.2	7±14
Others	31673.2±3255.6	27902.4±2388.4	29515.8±1562.6	27656.4±1432.7	30734.6±519.9

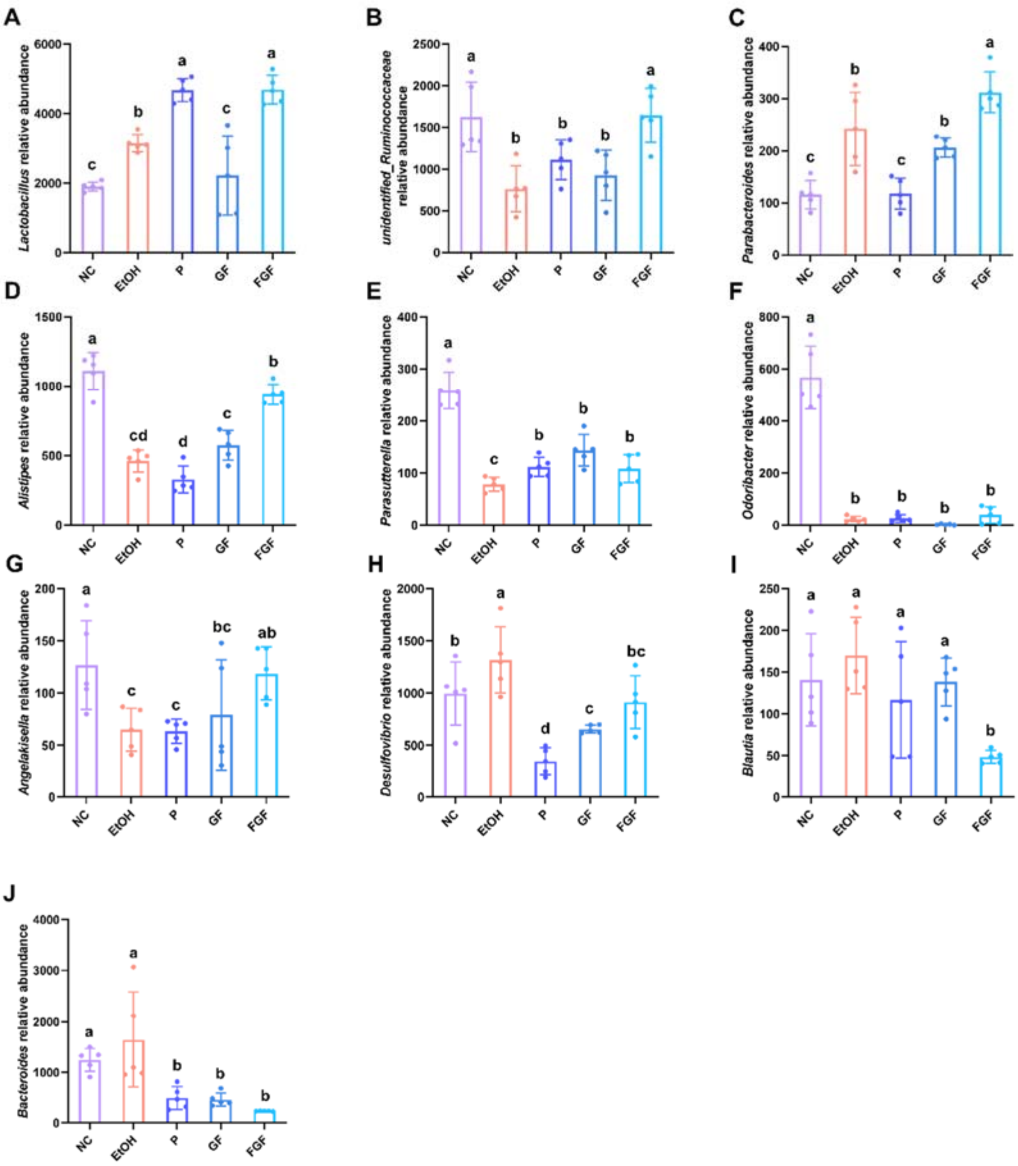

Figure S1. The relative abundance of key intestinal microbiota. Data are expressed as the mean ± SD (*n* = 5)
